# Comparative nutrient composition of selected wild edible mushrooms from two agro-ecological zones, Uganda

**DOI:** 10.1186/s40064-015-1188-z

**Published:** 2015-08-20

**Authors:** Immaculate Nakalembe, John David Kabasa, Deogratias Olila

**Affiliations:** Department of Biomolecular Resources and Biolaboratory Sciences, Makerere University, P. O. Box 7062, Kampala, Uganda; Department of Biosecurity, Ecosystems and Veterinary Public Health, Makerere University, P. O. Box 7062, Kampala, Uganda; Department of Pharmacy and Comparative Medicine, Makerere University, P.O. Box 7062, Kampala, Uganda

**Keywords:** Wild mushrooms, Nutritional attributes, Agro-ecological zones

## Abstract

In Uganda, wild mushrooms are mainly collected during the rainy season and valued as a traditionally nutritious food by the rural poor. However, their nutritional attributes have not been adequately studied and documented. Comparative nutrient composition of five wild edible mushroom species was determined, namely: *P. tenucuilus*, *T. tyleranus*, *T. clypeatus*, *V. speciosa* and *T. microcarpus* of sub-humid and humid agro-ecological zones. Standard analytical techniques following the AOAC were used for proximate and mineral contents determinations. Vitamins determination followed the established standard protocols of the laboratories where the analyses were conducted. Combined use of nutrient concentration and scores were used to compare the level of the contents in the mushroom species. Significant differences (p < 0.05) in nutrient values were demonstrated between and among the mushroom species obtained from the two agro-ecological zones. On dry weight basis, all proximate compositions were high in mushroom species obtained from the humid zone with exception of the total carbohydrates and energy values. Irrespective of the source of the mushrooms, significant amounts were demonstrated in protein, dry matter, ash and total carbohydrates ranging between 11.56–27.42%, 82.34–99.76%, 10.79–16.87%, and 37.12–61.05%, respectively. In comparison with recommended dietary daily intakes, the K, P, Se, Mn, Cu and Fe contents were relatively high with low Ca, Mg, Zn and Na. Thiamin, folic acid, vitamin C, and niacin levels were high but below the recommended FAO references. Considering mushrooms from different agro-ecological zones, significant differences (p < 0.05) were observed in all mushroom species in P except in *T*. *clypeatus*, *T. tyleranus, T. microcarpus* and *T. clypeatus* in potassium, *T. clypeatus* and *T. microcarpus* in Mg. Mushrooms from humid agro-ecological zones had relatively high overall mineral and vitamin supply potential. In conclusion, consumption of these mushrooms should be encouraged in supplementation of the staple food of the poor people. Hence, solving malnutrition problems in children, pregnant mothers, and the immune compromised patients such as the HIV/AIDs.

## Background

Over 2,500 different mushrooms grow in the wild around the world and their nutritional potential has long been overshadowed by the well known cultivated mushrooms such as *Lentinus edodes*, *Pleurotus species* and *Agaricus bisporus.* A high biodiversity of wild mushroom biota is found throughout the tropics, particularly sub-Saharan Africa, with about 300 edible mushroom species (Harkonen et al. 1995; Rammeloo and Walleyn 1993). Many of these fungi belong to the genus *Termitomyces*, with a symbiotic relationship with termites (Heim 1958). *Termitomyces* are said to be superior to all other mushrooms globally because of their biting aroma (Singer 1962). Several ethnic groups of tropical countries consume wild mushrooms (Chang and Mshigeni 2001; Van Dijk et al. 2003; Nakalembe et al. 2009). In Uganda, several mushroom species such as *Armillaria mellea*, *Lentinus prolifer*, *Termitomyces aurantiacus*, *Termitomyces eurrhizus* and *Termitomyces microcarpus* among many others grow in most parts of the country (Katende et al. 1999; Nakalembe et al. 2009). Most of these wild mushrooms are reportedly used for food, medicine, cultural festivals and to some extent for income generation (Nakalembe et al. 2009). However, their nutritional value has never been adequately studied and documented. The objective of this investigation was to determine and compare some nutrient composition of five mushroom species obtained from two agro-ecological zones of Mid-Western Uganda.

## Methods

### Mushrooms, source, collection and handling

Five edible wild mushrooms species were studied: *Termitomyces microcarpus* (Berk and Broom) R. Heim, *Termitomyces tyleranus* (Otieno), *Termitomyces clypeatus* (Heim), *Volvariella speciosa* (Fr. ex Fr.) Singer and *Polyporus tenucuilus* (Beauv.) Fr. Mushrooms were collected during the same rainy season (March–June, 2013) from humid and sub-humid agro-ecological zones, with their caps curved downwards before shedding off spores. Identification of the mushrooms was done both locally (at source) and macroscopic features of the mushrooms were examined at the College of Agricultural Sciences and Environmental Management, Department of Botany, Makerere University. On-site samples were prepared for DNA assays, whereby health fresh tissues of mushrooms were aseptically removed from the inner part of the caps, placed between clean papers, labeled and wrapped in aluminum foil. The necessary information concerning each mushroom species such as the habitat, substrate and any associations was sent together with the mushrooms for scientific identification at the Key Laboratory of Systematic Mycology and Lichenology, Institute of Microbiology, Chinese Academy of Sciences, China. Molecular identification was done by amplifying and sequencing the Internal Transcribed Spacer (ITS 4 and ITS 5) of the nuclear ribosomal DNA (nrDNA) of the mushrooms. The sequenced DNA fragments were compared with data set obtained from NCBI GenBank. Herbarium specimens were prepared and deposited in the Makerere University Herbarium.

### Analytical methods

All mushroom species were cleaned, air-dried, ground and transferred into polythene bags, labeled and sealed to prevent any moisture intake and then stored in a refrigerator at 4°C until required for analysis. Cold samples were allowed to attain room temperature before analysis was done.

### Proximate analysis

Three grams (3 g) of each ground air-dried mushroom species were analyzed for their proximate contents using AOAC (AOAC 2002) method. The moisture content was determined by air-oven drying at 110°C for 1 h, and the crude fiber (dilute acid and alkali hydrolysis), crude fat (acid hydrolysis), crude protein by microKjeldahl method (%Protein = N × 4.38) and ash (ashing at 550–600°C for 3 h). Each mushroom analysis was done in duplicates on dry weight basis. The contents of total carbohydrate was calculated as differences as follows: total carbohydrates (%) = 100 − [moisture (%) + crude fiber (%) + crude fat (%) + ash (%)]. Energy values (Kcal) were calculated using Crisan and Sands’ conversion factors (Crisan and Sands 1978).

### Mineral and vitamin determinations

For determination of the minerals and vitamins, 3 g of each air-dried mushroom sample was and analyzes done in duplicates. Mineral constituents comprising potassium (K), magnesium (Mg), iron (Fe), calcium (Ca), copper (Cu), manganese (Mn) and zinc (Zn) were determined by Atomic Absorption Spectrophotometry (Unicam Analytical system, Model 919, Cambridge, UK), whereas phosphorus (P) and sodium (Na) determination were determined using a flame photometer (Jenway, PF 7, Essex UK). For selenium (Se) analysis, digestion was done using an advanced Microwave digestion system (Milestone ETHOS, Labstation with easyWAVE) with nitric acid and hydrogen peroxide, and then analyzed using an inductively coupled plasma (ICP) spectrometer (iCAP 6000 series; Thermo Scientific). All minerals were expressed in g/100 g dry weight basis, except selenium (µg/100 g).

Vitamins were determined using standard procedures. Ascorbic acid was determined by Iodine Titration (Helmenstine 2001), pantothenic acid by an AOAC Official Method 945.74, biotin by a method based on the USP 31 and vitamin B_12_ determined by vitamin B_12_ assay SOP No.: 18/1.3 by Ramula. A. of analytical division, Lifescience facility, India. Alpha-tocopherol analysis was carried out according to Delgado-Zamarreño et al. (2001) and vitamin A determined as β-carotene using AOAC Official Method 971.15 of spectrophotometric method. Water-soluble vitamins (B1, B2, B3 and folic acid) were measured with a RIDASCREEN enzyme immunoassay kit (R-Biopharm AG, Darmstadt, Germany) in accordance with the manufacturer’s instructions.

### Mineral and vitamin scorings

Mineral and vitamin scorings were done according to Kabasa et al. (2004). The mineral contents were transformed into mineral or vitamin scores basing on the following formulae in order to ease interpretation of mineral composition:

1 $${\text{Mushroom IMS/IVS}} = \left( {\frac{\text{Mushroom mineral or vitamin content}}{\text{Recommended dietary mineral or vitamin content}}} \right) \, \times { 100}$$

2 $${\text{Mushroom SMS/SVS}} = \sqrt[z]{{\prod\limits_{i = 1}^{z} {IM/VS_{i} } }}$$

where individual mineral or vitamin score (IMS/IVS) is the score of a specific mineral or vitamin nutrient in a mushroom species, whereas species mineral/vitamin score (SMS/SVS) is the score of the mushroom species for all its minerals/vitamins combined and is the geometric mean of IMS. z is the number of terms or specific mineral or vitamin nutrients evaluated in the expressions. Individual mineral or vitamin scores at the recommended dietary intake were similarly transformed into high, adequate or deficient species scores. In all cases, dietary nutrient recommendations of the Food and Nutrition Board, Institute of medicine (2001, 2004) were adopted.

### Data analysis

Data was subjected to analyses of variance (one-way ANOVA) using the Statistical Package for Social Sciences (SPSS) Program 10.1 version. Significance was accepted at the 5% probability level. An independent sample *t* test was used to compare nutrient values from different agro-ecological zones and results were given as mean ± standard deviation (SD) of two replicates.

## Results

The proximate composition and energy values of the wild edible mushrooms are shown in Table 1. Significant differences (p < 0.05) were observed in all proximate composition and energy values of corresponding mushroom species except in *P. tenuiculus* in ash, *V. speciosa* in dry matter and moisture, *T. microcarpus* in crude lipid, *T. tyleranus* in crude fibre and *T. clypeatus* in ash contents. Compared to other mushroom species, *T. microcarpus* and *T. tyleranus*, irrespective of their source, had significantly high crude protein, carbohydrates, crude fiber and with low energy values, whereas *P. tenuiculus* and *T. clypeatus* exhibited low ash, crude protein, nitrogen and high crude fibre and energy values. Significant differences (p < 0.05) were also observed between mushrooms from the same agro-ecological zones.

**Table 1:** Proximate composition (%) of priority wild mushroom species

Treatment	AE	*Volvariella speciosa*	*Polyporus tenuiculus*	*Termitomyces microcarpus*	*Termitomyces tyleranus*	*Termitomyces clypeatus*
Ash	H	14.13 (0.12)^a^	11.65 (0.24)^b^	15.13 (0.07)^a^	16.87 (0.01)^a^	11.2 (0.10)^b^
	Sh	11.65 (0.04)^b^	12.24 (0.37)^b^	13.82 (0.65)^b^	13.68 (0.89)^b^	10.79 (0.02)^b^
Dry matter	H	86.02 (0.30)^a^	83.25 (0.16)^a^	83.01 (0.15)^a^	84.63 (0.66)^a^	82.34 (0.24)^b^
	Sh	85.6 (0.08)^a^	87.70 (0.91)^b^	86.96 (0.13)^b^	89.76 (0.63)^b^	85.87 (3.05)^a^
Crude protein	H	19.95 (0.39)^b^	16.86 (0.38)^a^	27.42 (0.22)^c^	21.77 (0.003)^d^	18.00 (0.32)^b^
	Sh	13.84 (0.26)^a^	11.56 (0.26)^b^	13.00 (0.71)^a^	14.59 (0.26)^a^	12.76 (1.4)^a^
Crude fiber	H	4.19 (0.12)^c^	9.46 (0.82)^b^	3.95 (0.50)^c^	3.08 (0.13)^a^	7.69 (0.13)^d^
	Sh	8.57 (0.22)^b^	5.20 (1.13)^c^	2.08 (0.64)^d^	2.80 (0.52)^a^	3.79 (0.28)^e^
Crude lipid	H	3.56 (1.05)^a^	3.23 (0.19)^a^	3.34 (0.21)^a^	3.00 (0.02)^a^	3.79 (0.28)^a^
	Sh	2.81 (0.04)^a^	2.85 (0.99)^a^	2.50 (0.5)^a^	2.02 (0.05)^b^	2.24 (1.07)^c^
Moisture	H	13.98 (0.3)^a^	16.75 (0.2)^b^	16.99 (0.15)^b^	15.37 (0.85)^b^	17.66 (0.24)^b^
	Sh	12.40 (0.08)^a^	12.30 (1.85)^a^	13.04 (0.12)^a^	10.24 (0.63)^c^	14.13 (3.05)^a^
Nitrogen	H	4.45 (0.05)^b^	3.85 (0.001)^b^	6.26 (0.03)^c^	4.97 (0.05)^b^	4.11 (0.07)^b^
	Sh	3.16 (0.07)^a^	2.64 (0.16)^a^	2.97 (0.06)^a^	3.33 (0.32)^a^	2.91 (0.06)^a^
Total carbohydrate	H	48.44 (0.06)^a^	51.51 (0.17)^c^	37.12 (0.76)^b^	42.99 (0.11)^e^	49.35 (0.41)^a^
	Sh	59.3 (1.38)^b^	61.05 (1.14)^d^	57.64 (0.58)^d^	59.47 (0.78)^d^	60.08 (5.8)^d^
Energy (kcal)	H	248.64 (0.12)^a^	250.46 (0.10)^a^	228.97 (0.10)^c^	220.75 (0.82)^c^	250.62 (0.20)^a^
	Sh	266.14 (0.01)^b^	266.60 (0.50)^b^	255.57 (0.62)^b^	263.08 (1.09)^b^	261.26 (0.71)^b^

Figures in parentheses are standard deviations of means.

^a,b,c,d^ Values along the rows and between agro-ecological zones are significantly different at p < 0.05. Mean ± SD of two replicates. Crude protein = N × 4.38, carbohydrates by difference.

*H* humid, *S* sub-humid, *AE* agro-ecological zone.

Irrespective of the mushroom source, P and K contents were high and low in Mg, Ca and Na (Table 2). Considering mushrooms from different agro-ecological zones, significant differences (p < 0.05) were observed in all mushroom species except T. *clypeatus* in P, *T. clypeatus* and *T. microcarpus* in Mg; *T. tyleranus* and *T. clypeatus* in K and Ca in *Volvariella speciosa*. Variations were also obtained within mushrooms of the same agro-ecological zone. Mineral deficiencies were more pronounced for major but not for trace minerals.

**Table 2:** Major mineral contents (g/100 g dry basis) and scores of wild edible mushrooms

Ms	AE	Potassium	IMS	Sodium	IMS	Calcium	IMS	Magnesium	IMS	Phosphorus	IMS
1	H	2,530.1 (964)^a^	67	16.8 (8.48)	0.7	15.5 (4.03)	1.6	31.9 (29.9)	8.0	794.9 (0.1)	80
	Sh	2,514.9 (955)	66	10.2 (4.46)	0.4	13.2 (2.2)	1.3	25.3 (8.29)	6.3	508 (2.83)	51
2	H	3,354.4 (12.5)	88	11.4 (0.64)	0.5	13.4 (0.77)	1.3	30.58 (0.75)	7.6	564.6 (2.9)	56
	Sh	2,131.4 (71)	56	14.5 (0.07)	0.6	10.6 (0.52)	1.1	13.13 (0.91)	3.3	686.5 (0.2)	69
3	H	1,869.7 (214)	49	10.3 (14)	0.4	14.8 (0.74)	1.5	10.32 (0.35)	2.6	612.3 (0.2)	61
	Sh	1,809.7 (13.9)	48	10.4 (0.8)	0.5	11.9 (0.15)	1.2	10.19 (0.25)	2.5	616 (7.071)	62
4	H	1,954.4 (107)	51	12.6 (1.7)	0.5	13.8 (0.7)	1.4	13.77 (2.86	3.4	840.5 (0.69)	84
	Sh	2,212 (385)	58	16.6 (11.0)	0.7	12.3 (0.47)	1.2	13.05 (2.52)	3.3	512.5 (3.54)	51
5	H	3,196.4 (450)	81	16.1 (8.06)	0.7	12.8 (2.12)	1.3	7.14 (1.44)	1.8	612 (4.24)	61
	Sh	2,939.1 (385)	77	15.8 (0.00)	0.7	13.8 (1.18)	1.4	9.18 (0.00)	2.3	782 (4.24)	78

Mean ± SD of two duplicate. IMS represents individual mineral score of the mushroom species measured at recommended daily intake for physiological needs recommended by FND 2001. <100 score indicates lower mushroom mineral content; >20 score indicates relatively high levels of mineral content in a serving; 10-19 score indicates good mineral content in a serving; <10 indicates poor source of that particular mineral in a serving.

*AE* agro-ecological zone, *H* humid, *Sh* sub-humid.

^a^Figures in parentheses are standard deviations of means; *Ms* mushroom species; 1, *Termitomyces tyleranus*; 2, *Polyporous tenuiculus*; 3, *Termitomyces clypeatus*; 4, *Termitomyces microcarpus*; 5, *Volvariella speciosa.*

**Fig. 1:**
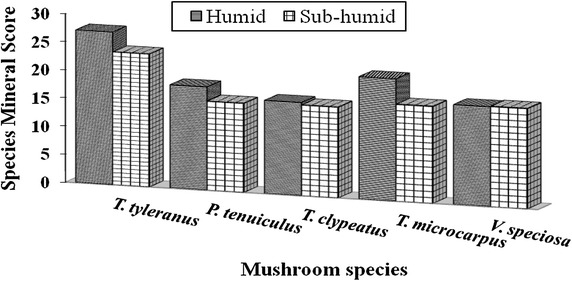
Species mineral scores (SMS). Species mineral scores were calculated as geometric means of individual mineral scores for each mushroom species. A *lower mineral score* indicates a lower overall mineral supply potential of the mushroom species.

Among major elements, deficiencies were highest for Na followed by Ca and Mg, and Zn for trace elements. Among the trace elements, Se content was high followed by Fe, Mn and Cu, with very low Zn contents (Table 3). Significant differences (p < 0.05%) of these minerals were observed in all mushroom species from different agro-ecological zone except *T. clypeatus* in Se, Mn and Zn contents. Species mineral scores (Fig. 1) showed significant differences (p < 0.05) except *P. tenuiculus* and *T. clypeatus.* Mushrooms from humid zone had high SMS than its counterpart. Irrespective of the source, *T. tyleranus* had the best SMS followed by *T. microcarpus,* respectively. Mushroom species from the humid agro-ecological zones exhibited high nutrient supply potential as shown by the species nutrient scores.

**Table 3:** Trace mineral content and scores of wild edible mushrooms

Mushroom species	AE	Selenium (µg/100 g)	IMS	Copper (mg/100 g)	IMS	Manganese (mg/100 g)	IMS	Iron (g/100 g)	IMS	Zinc (g/100 g)	IMS
*Termitomyces tyleranus*	H	125 (0.4)	179	0.8 (31.1)	40	1.19 (4.24)	60	22.7 (62.3)	126	1.1 (5.37)	7
	Sh	130 (2.1)	186	0.23 (3.7)	12	0.64 (7.50)	32	18.2 (10.2)	101	0.94 (6.7)	6
*Polyporous tenuiculus*	H	108 (0.9)	154	0.35 (3.1)	18	0.46 (1.77)	23	13.3 (10.6)	74	0.78 (0.4)	5
	Sh	112 (0.01)	160	0.28 (2.5)	14	0.35 (1.77)	18	11.1 (1.56)	62	0.57 (0.0)	4
*Termitomyces clypeatus*	H	119 (0.5)	170	0.23 (3.3)	12	0.58 (14.9)	29	13.3 (6.2)	74	0.75 (1.2)	5
	Sh	120 (3.2)	171	0.25 (0.6)	13	0.55 (1.77)	29	11 (2.83)	61	0.75 (1.3)	5
*Termitomyces microcarpus*	H	142 (0.4)	203	0.7 (17.9)	35	0.97 (6.58)	49	21.8 (2.19)	121	0.76 (5.8)	5
	Sh	146 (1.8)	209	0.23 (1.7)	12	0.78 (6.72)	39	13 (1.2)	72	0.56 (0.8)	4
V*olvariella speciosa*	H	148 (4.6)	213	0.21 (1.2)	11	0.83 (7.95)	42	13.8 (4.03)	77	0.61 (3.7)	4
	Sh	137 (0.7)	196	0.26 (1.9)	13	0.25 (3.25)	13	15.1 (11.3)	83	0.74 (5.7)	5

Mean ± SD of duplicates.

Figures in parentheses are standard deviations of means; *IMS* individual mineral score of the mushroom species measured at recommended daily intake for physiological needs of FND (2001). <100 score indicates deficient mineral content; > 20 score indicates relatively high mineral content in a serving; 10–19 score indicates good source of that particular mineral in a serving; < 10 indicates poor mineral level in a serving.

**Fig. 2:**
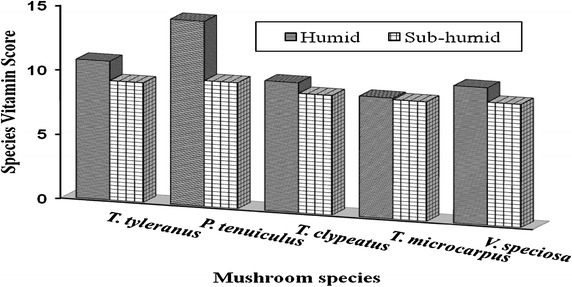
Species vitamin scores of individual mushroom species. Species vitamin scores were calculated as geometric means of individual vitamin scores for each mushroom species. A *lower vitamin score i*ndicates a lower overall vitamin supply potential of the mushroom species.

The vitamin contents in mushrooms were both species and source-dependent (Table 4). Significant differences (p < 0.05) in vitamin contents were recorded within and between the agro-ecological zones. The mushrooms were rich in folic acid followed by niacin, vitamin C and thiamin, and low in riboflavin, β-carotene and α-tocopherol as compared to the recommended daily intakes. Pantothenic acid, biotin and vitamin B_12_ were not detected in all mushroom species. Alpha-tocopherol was not identified in *T. globulus.* Individual vitamin scores (IVS) indicate deficiency of riboflavin, β-carotene and α-tocopherol, with high folic acid, vitamin C, niacin and thiamin concentrations (Table 4). Generally, mushrooms obtained from humid agro-ecological zone had moderately good species vitamin scores (Fig. 2).

**Table 4:** Average vitamins content and their individual scores of edible wild mushrooms

Mushroom species	AE	Thiamin (mg/100 g)	IVS	Riboflavin (mg/100 g)	IVS	Niacin (mg/100 g)	IVS	Folic acid (μg/100 g)	IVS	β-carotene (μg/g)	IVS	α-Tocopherol (μg/100 g)	IVS	Vitamin C (mg/100 g)	IVS
*Polyporus tenuiculus*	H	0.94 (0.02)^a^	63	0.09 (0.02)^a^	5	6.95 (0.3)^a^	35	158.5 (2.1)^a^	40	14.7 (0.04)^a^	4.8 mg/day^A^	0.43 (0.02)^a^	4	18.1 (0.4)^b^	24
	Sh	0.35 (0.01)^b^	23	0.07 (0.01)^b^	4	3.86 (0.04)^b^	19	99 (1.41)^b^	25	13.6 (0.22)^a^		0.44 (0.1)^a^	4	13.1 (0.5)^a^	18
*Termitomyces microcarpus*	H	0.17 (0.01)^c^	12	0.06 (0.01)c	4	3.46 (0.01)^b^	17	120 (0.00)^c^	30	16.6 (0.02)^b^		0.46 (0.01)^a^	5	13.57 (0.1)^a^	18
	Sh	0.79 (0.01)^d^	5	0.06 (0.03)c	4	5.29 (0.01)^c^	26	160 (1.4)^a^	40	17.4 (0.25)^b^		0.51 (0.24)^a^	5	14.07(0.1)^a^	19
*Termitomyces tyleranus*	H	0.35 (0.00)^b^	23	0.07 (0.01)^a^	4	7.31 (0.01)^b^	37	70(0.00)^c^	18	15.0 (0.00)^a^		0.52 (0.02)^a^	5	14.37 (0.1)^a^	19
	Sh	0.18 (0.02)^c^	12	0.09 (0.00)^b^	5	2.15 (0.00)^d^	11	120 (0.71)^a^	30	14 (0.17)^a^		0.51 (0.01)^a^	5	14.57 (0.1)^a^	19
*Termitomyces clypeatus*	H	0.19 (0.01)^c^	13	0.05 (0.03)^c^	3	7.3 (0.01)^a^	37	150 (0.04)^a^	38	12.6 (0.02)^c^		0.45 (0.00)^a^	5	15.1 (0.1)^a^	20
	Sh	0.05 (0.8)^d^	4	0.06 (0.02)^c^	4	2.13 (0.01)^d^	11	380 (0.35)^e^	95	17.7 (0.01)^a^		0.45 (0.01)^a^	5	17.8 (0.02)^b^	24
*Volvariella speciosa*	H	0.24 (0.04)^e^	16	0.06 (0.01)^b^	4	2.66 (0.13)^c^	13	159 (0.01)^a^	40	15.2 (0.04)^b^		ND	0	11.05 (0.8)^c^	15
	Sh	0.14 (0.01)^f^	9	0.08 (0.02)^b^	5	2.85 (0.02)^c^	14	87.1 (0.01)^b^	22	13.5 (0.34)^a^		ND	0	21.40 (0.4)^d^	29

Individual vitamin scores (IVS) of wild mushrooms were measured at recommended daily intake for physiological needs (FND 2003). >20% and below 100 score indicates high vitamin content in a serving; 10–19 score indicates good vitamin content in a serving; <10 indicates low vitamin content in a serving.

Figures in parentheses are standard deviations of means.

NB: Pantothenic acid (ng/g), biotin (ng/g) and vitamin B_12_ (µg/g) were not detected at their lowest concentration.

*IVS* Individual Vitamin Scores, *ND* not detected, *AE* agro-ecological zone, *Sh* sub-humid, *H* humid.

^A^Recommended daily intake for β-carotene not known.

^a,b,c,d^ Values with different superscripts within a row and between agro-ecological zones are significantly different (p < 0.05).

## Discussion

The substrate, mushroom species, age and part of the basidiomata as well as mushroom storage conditions after harvest are one of the factors that affect mushroom chemical composition (Adejumo and Awosanya 2005). More still, results of the same species of mushrooms obtained by different investigators may be different due to the influence of different techniques of analysis involved. Therefore, the data generated by other investigators can only be used to produce estimates of probable nutritive value of given mushroom species.

Generally, this study indicates that the studied mushrooms are good sources of proteins, crude fiber, carbohydrates, vitamins and minerals. Mushrooms from humid zone had high concentration of nutrients, which may have been contributed by the acidity and organic matter content of the soil (Colak et al. 2009). Furthermore, different species of mushrooms had variable amount of nutrients probably because of species/strain differences and their ability to bioaccumulate the minerals and other nutrients into their tissues (Mattila et al. 2001; Mshandete and Cuff 2007).

High dry matter contents observed in these mushrooms is due to lowered moisture contents, which would otherwise affect the concentration of soluble solids that make up the dry matter. Moisture variation may have been caused by a series of factors such as the environmental factors during growth and storage; and the relative amount of metabolic water produced during storage (Mattila et al. 2001). The moisture and dry matter values were comparable with previously reported data in Uganda (Opige et al. 2006; Kabasa et al. 2006).

In this study, large amounts of total carbohydrates are contained in the dry matter of mushrooms, which is consistent with the values reported for wild mushrooms in other parts of the world (Sanmee et al. 2003; Gbolagade et al. 2006; Saiqa et al. 2008). It constitutes of the chitin, polysaccharides (β-glucans) and sugar alcohols (Kurtzman 1997; Mattila et al. 2001). A considerable amount of this carbohydrate is dietary fiber that would serve as roughage with low calorific value. The presence of sugar alcohols makes these mushrooms substitutes for the high energy sugar in diabetic patients (Hamano 1997).

Bernaś et al. (2006) observed that differences in protein contents could be due to species/strain specific, the growth substrate, pileus size, time of harvest and the level of nitrogen available in the growth substrate. The content of protein varied between 17.58 and 27.43% on a dry weight basis for *P. tenucuilus* (sub-humid) and *T. microcarpus* (humid region) making up to 35.2–54.9% of 50 g of protein/day of Recommended Daily Intake (RDI), which is within the average value of 19–35% dry weight for dried mushrooms (Crisan and Sands 1978). These results are in accordance with those in literature (Sanmee et al. 2003), and well below other documented values of some wild mushrooms (Adejumo and Awosanya 2005; Chye et al. 2008; Colak et al. 2009; Palazzolo et al. 2012). As compared to the staple foods; 7.3% rice, 12.7% wheat, 9.4% maize, these mushrooms contain much more protein, and therefore, mushroom protein can supplement the low protein diets of poor people who cannot afford animal resource foods.

The total fat content was low, varied from 2.015 to 3.79% dry weight (3.1–5.83% RDI of 65 g of crude fat/day). Mushrooms obtained from humid areas had the highest fat content 2.995–3.79%. However, the fat content was within the range of the reported value of wild mushrooms i.e. 1.1–8.1% on dry weight basis (Crisan and Sands 1978), and slightly lower than wild mushrooms of Eastern Uganda (3.868–4.491% dry weight) (Opige et al. 2006). In this study, crude fat content is comparable to that of millet (2.8%) and maize (4.2%) (FAO 1972). Mushroom fat is reportedly low but rich in essential unsaturated fatty acids which are considered essential for human diet and health.

Crude fiber is also part of a healthy diet. It was within the reported value of 3–35% fiber on a dry weight basis (Breene 1990) and ranged between 2.084–9.46% (8.34–37.84% RDI, *25 g of fiber/day). Compared with cereals and vegetables, mushrooms are as good as cereals but with high fiber value compared to some vegetables e.g. carrots (0.6%) and lettuce (0.2%) (FAO 1972). The results of this study concur with crude fiber values of cultivated mushroom species elsewhere, for instance, 7.3–9.3% for *Coprinus*, 7.5–16.3% for *Pleurotus* (La Guardia et al. 2005) and 5.5–17.4% for *Volvariella* species (Aletor 1995). However, Chye et al. (2008) recorded high crude fiber content of some of the wild mushrooms such as *P. tenuiculus* (39.85%). Since the mushroom species examined contained significant amounts of crude fiber, they could be regarded as good sources of dietary fiber for supplementation of some foodstuffs with less fiber such as vegetables, hence utilized as roughage, and mostly its immune-stimulation effects should not be overlooked.

Energy values of the studied wild mushrooms were low and dependant on both mushroom species and the source (Table 1). They varied from 220.75 to 266.14 kcal/100 g dry weight providing only 11.04–13.31% of the RDI of 2,000 kcal/day. The energy values are slightly below that of cereals (millet 341 kcal and maize 349 kcal) (FAO 1972). Other studies done elsewhere reported relatively high energy values of wild mushrooms ranging from 367.9–450.2 kcal/100 g (Mshandete and Cuff 2007; Colak et al. 2009). Taking this into consideration, mushrooms can be used for weight management.

Like many other organisms, mushrooms bioaccumulate several minerals from their growth media with the help of its greater part, the mycelia. The latter has the highest growth rate, is compressed in nature and is spread over areas of several square metres (Eurola et al. 1996). These facilitate its efficiency extraction of nutrients from the growth media and bioaccumulate them in relative quantities. However, mineral concentrations in fungi may depend on several interdependent factors namely the climate, season of the year, state of maturity, basidiomata part as well as storage conditions after harvest (Varo et al. 1980; Adejumo and Awosanya 2005).

Ash in edible fungi ranges from 5 to 13 g/100 g dry matter (Varo et al. 1980) with the major minerals constituting about 56–70% of the total ash content (Li and Chang 1982). Potassium accounts for nearly 45% of the total ash content. In this study, the ash content of mushrooms ranged between 11.2 and 16.9% for humid areas while 10.79–13.82% for sub-humid. Other studies reported relatively higher ash content (Wardlaw and Kessel 2002) while others very low as small as 2.45% for *P. tenuiculus* and 2.00% for *Lycoperdon perlatum* (Colak et al. 2009). The wild mushrooms under study were more deficient in major minerals than trace elements especially in Na, Ca and Mg. Potassium and phosphorus were the predominant elements among the major minerals. This is in line with other studies done elsewhere in the world (Mattila et al. 2001; Barros et al. 2008; Chye et al. 2008; Colak et al. 2009; Palazzolo et al. 2012). Potassium ranged from 1809.7 to 3354.45 mg/100 g making 38.4–71.4% RDI of 4,700 mg/day and P ranged from 508 to 840 mg/100 g (51–84% RDI, 1,000 mg/day). The contents of K were high compared to Na; an important attribute from the nutritional point of view as it is a main electrolyte and major cation inside the cell. Phosphorus content was high in contrast to some Nigerian wild mushrooms (2.3–29.8 mg/100 g; Gbolagade et al. 2006) and very low in *T. microcarpus* of Uganda (156.53 mg/100 g; Nabubuya et al. 2010) on dry weight basis. Phosphorus is essentially required by all cells in the body for normal functioning (Knochel 1999). It is efficiently absorbed from the gastrointestinal tract and available in most foods, hence less important in diet planning (Wardlaw and Kessel 2002).

Magnesium (7.14–31.9 mg/100 g) was low contributing about 1.8–8.0% of RDI of 400 mg/day. Magnesium was found to be as low as 0.5–1.6 mg/100 g dry weight in ectomycorrhizal mushrooms (Sanmee et al. 2003) on dry weight basis. However, their contents in this study are slightly low in some wild mushrooms (0.4–6.7 mg/100 g, Gbolagade et al. 2006) but too low compared to green vegetables, legumes and whole grain cereals (100–500 mg/100 g), meat and dairy products (100–300 mg/100 g) (FAO 1972). Therefore, this requires a combination of the latter foods together with these mushrooms in order to obtain adequate magnesium for proper functioning of the body.

Furthermore, Ca and Na levels were also quite low in these mushrooms contributing only 1.1–1.6% and 0.4–0.7%, respectively of the RDIs. In contrast, the sodium contents reported elsewhere are as low 0.28 mg/100 g dry weight basis (Mattila et al. 2001). However, low Na concentrations are of great benefit nutritionally to the consumer, especially in hypertensive patients (Feldman et al. 1986). Mattila et al. (2001) reported insignificant levels of Ca in cultivated mushrooms ranging from 0.05 to 0.25 g/kg dry weight, and very low in ectomycorrhizal mushrooms (Sanmee et al. 2003). Calcium content (10.6–15.5 g/100 g) in this study was in agreement with other studies (Gbolagade et al. 2006), higher than in other studies carried out in Uganda (0.0095–0.0115%, Olila et al. 2008) and very low compared to other values (77–144.7 mg/100 g) elsewhere in the world (Chye et al. 2008). However, it was comparable to many common vegetables (10.0–52.0 mg/100 g) (FAO 1972), and lower than the recommended daily intakes in all age groups (Food and Nutritional Board 2001, 2004). Its low content in mushrooms suggests a low intake of Ca in vegetarians; therefore supplementation is required to meet their Ca requirements.

Among the trace elements, Se content was higher (120–148 µg/100 g) than other trace elements, as well as the recommended FAO daily intake (55 µg/100 g). Sanmee et al. (2003) reported Se contents of ectomycorrhizal mushrooms as extremely low to very high (0–12,600 µg/100 g); whereas cultivated mushrooms had a value between 0.33 and 320 µg/100 g. About 70 and 50 µg of Se were suggested as sufficient daily requirement for men and women, respectively (Food Nutrition Board 2000); whereas Yang et al. (1988) proposed 600 and 400 µg as the daily maximum and safe intake of dietary selenium, respectively. This proves that the studied mushrooms are safe for human consumption. Selenium is an essential nutrient required for prevention and treatment of several conditions including cardiomyopathy, fatigue and keep the keratinized tissues normal (Kumar 1995) in addition to being a powerful antioxidant (Charanjeet et al. 2003).

Iron content (10.6.6–22.7 g/100 g) was above the RDI (18 mg/day) as well as the upper level intake (40–45 mg/day) (Food and Nutrition Board 2001), higher than the published value for cabbage (6 mg/kg) and that of those sources considered nutritionally dense in Fe such as meat (16 mg/kg) (Eyabi 2001). The Fe content is quite higher than the tolerable upper intakes (40–45 mg/day). Iron content in these wild mushrooms is line with other studies done on wild mushrooms (Zakhary et al. 1983; Chye et al. 2008; Colak et al. 2009), but higher than in other studies (0.07–0.09 mg/100 g, Gbolagade et al. 2006). However, this total Fe content may not constitute an accurate guide to confirm the levels in this resource, but its bioavailability, which also depends on its form, the needs of the body and the presence or absence of antinutritional factors that may influence its absorption. However, it was observed that mushrooms contain insignificant levels of phytates, which would otherwise affect its bioavailability (Colak et al. 2009). Despite this, one should take care when consuming mushrooms in combination with other foods such as vegetables that may influence absorption of Fe.

Fe was followed by Mn ranging from 0.25 to 1.19 mg/100 g (12.5–60% RDI, 2 mg), a value within that of bread and cereals (0.68–9 mg/kg) and slightly lower than in eggs, milk and meat (<1 mg/kg); and green vegetables (2 mg/kg) (Pelkonen et al. 2008). Mn content in this study concur with other studies (Gbolagade et al. 2006), but it can be as high as 14.3 mg/100 g (Colak et al. 2009). Pelkonen et al. (2008) recommended 12 mg/day as an acceptable intake for the general public, suggesting that consumption of these mushrooms may not cause any intoxication to humans. Manganese is an important element in the body as it is a cofactor of enzymes and an antioxidant.

Copper is the third most abundant trace mineral in the body, and helps protect the cardiovascular, skeletal, and nervous systems. It ranged between 0.2 and 0.75 mg/100 g of copper, a value lower than the recommended daily intake (2 mg/day). However, this value is adequate for all age groups, except in pregnancy and lactation which require 1 mg/100 g of Cu (Food and Nutritional Board 2001). In contrast, other studies in Uganda indicated high copper content (Kabasa et al. 2006; Nabubuya et al. 2010) as well as in other parts of the world (Chye et al. 2008; Colak et al. 2009).

Zinc content was low in all mushroom species ranging from 0.56 to 1.1 mg/100 g (3.7–7.3% RDI, 15 mg), hence, very low for all age groups (Food and Nutrition Board 2001). Zinc concentrations were lower than in most meats (10–50 mg/kg), whole grain cereals (10–20 mg/kg) and milk (3 mg/100 g) (Pelkonen et al. 2008). Cellular metabolism involving immune function, protein synthesis, wound healing, DNA synthesis and cell division requires zinc (Heyneman 1996).

Vitamins contributes a very small percentage of food in our diet daily, but important in prevention of diseases and longevity (Olaniyi 2000). In this study, the wild edible mushrooms exhibited a good profile of vitamins, particularly thiamin, niacin, folic acid and vitamin C, as it was observed in other studies elsewhere (Mattila et al. 2001; La Guardia et al. 2005). Pantothenic acid, biotin and vitamin B_12_ were not detected in any of the mushroom species that were studied. Alpha-tocopherol was also not identified in *T. globulus.* The vitamin contents in this study were lower than what was obtained in fresh wild mushrooms (Chye et al. 2008); suggesting losses of these vitamins during mushroom handling such as drying. Vitamin concentrations in mushrooms may also be affected by several factors such as climatic conditions, strain of mushroom, stage of harvest, storage and handling of the mushroom samples during analysis.

The mushrooms contained high folic acid content, an essential vitamin in the maintenance of good health, treatment and prevention of anemic diseases (Refsum et al. 1998). Folic acid is one of the commonest deficiencies seen worldwide. It has been found to affect several groups of people including women on oral contraceptives, pregnant mothers, elderly, alcoholics and children are affected by its deficiency. In this study, folic acid content (70–380.3 µg/100 g) is adequate for all age groups except in pregnancy and lactation (500–600 μg/day) (Food and Nutritional Board 2001). This value is almost of the same magnitude as that generally found in some vegetables, higher than that of fruits (3–23 μg/100 g) (FAO 1972) and lower than in other wild mushrooms (1,222–1,412 μg/100 g dry weight) (Bano and Rajarathnam 1988). Fortunately, folic acid bioavailability in mushrooms is good compared to some vegetables, such as peas and spinach (Clifford et al. 1991).

Vitamin C contents were in moderate amounts (11.05–21.40 mg/100 g), contributing 14.7–28.5% of the recommended FAO/WHO (2005) daily intake of 75 mg/day. This value is adequate for children and very low for other groups of people (Food and Nutritional Board 2001). Due to its antioxidant and therapeutic properties, vitamin C is a valuable food component (Bernaś et al. 2006). The results of this study were quite higher than the range of the reported values in other mushroom species (Lau et al. 1985). However, vitamin C contents in these mushrooms concur with other reports elsewhere (Barros et al. 2008); and comparable to that of fruits and vegetables (FAO 1972). In contrast, Opige et al. (2006) found no detectable vitamin C in *T. microcarpus.* Although ascorbic acid contents varied considerably among the five types of wild edible mushrooms analyzed, these mushrooms could serve as a source of vitamin C in the diet.

These mushrooms showed considerable amounts of niacin (2.13–7.31 mg/100 g), providing only 10.7–36.6% of the recommended daily value (20 mg/day). The niacin value is within the RDI for children, but very low for other groups of people ((Food and Nutritional Board 2001). The mushrooms had high niacin contents comparable to eggs (0.8–4.4 mg/100 g), but lower than in meat (7.5–11.6 mg/100 g). These values are higher than those in vegetables and fruits (FAO 1972).

Thiamin content ranged between 0.05 and 0.94 mg/100 g (3.47–62.7%, RDI of 1.5 mg/day), relatively higher than those contained in fruits (0.02–0.07 mg/100 g) and eggs (trace-0.04 mg/100 g) but comparable to vegetables (0.01–0.12 mg/100 g), cereals (0.29–0.33 mg/100 g) (FAO 1972) and cultivated mushrooms (Mattila et al. 2001). These mushrooms provide adequate thiamin content to all groups of people except in pregnancy and lactation (Food and Nutritional Board 2001). Riboflavin content was low and ranged between 0.06 and 0.09 mg/100 g, making only 2.9–5.3% of the RDI (1.7 mg/day). It was slightly higher than that of fruits (0.01–0.05 mg/100 g, FAO 1972) but within the range of other mushrooms (Furlani and Godoy 2008), common vegetables (0.01–0.3 mg/100 g), and most common cereals (0.11–0.18 mg/100 g) (FAO 1972).

Beta carotene content was low (12.60–17.70 μg/g dry weight) but within the range of other reported ß-carotene values in wild mushrooms (2.52–75.48 µg/g dry weight, Barros et al. 2008). Chye et al. (2008) reported higher concentration of ß-carotenes in Malaysia’s wild mushrooms ranging from 0.37 to 2,711 μg/g fresh weight suggesting a loss of this vitamin during drying. However, cultivated mushrooms are reported to have lower concentrations (7.24 μg/g) than the mushrooms in this study. β-carotene is an important antioxidant required for stimulating the growth of new skin cells (Shukkit-Hale et al. 2006; Elmastas et al. 2007).

In addition to ß-carotene, these mushrooms were found to contain trace amounts of α-tocopherol ranging from 0.39 to 0.52 μg/100 g dry weight. Alpha tocopherol is the most active component of the vitamin E complex and a powerful antioxidant of the human body (Burton and Ingold 1989). The mushrooms lacked biotin, vitamin B_12_ and pantothenic acid, which observation is in disagreement with other reports elsewhere (Breene 1990; Mattila et al. 2001).

## Conclusion

Generally, the five mushroom species studied irrespective of their sources are good sources nutrients and compare favorably with the most common green leafy vegetables, legumes, whole grain cereals, meats and dairy products. In Uganda and indeed in other developing countries, micronutrient deficiency is the main cause of death and poor performance due to the poor staple foods consumed. This affects mainly the children, women, HIV-AIDs patients and the elderly. Therefore, if these mushrooms are encouraged to be consumed together with the common staple foods, especially of a poor man’s diet, stand a high chance of solving malnutrition in the above mentioned vulnerable groups of people.
